# PM_10_ Filter Monitoring and Moss-Bag Biomonitoring as Complementary Approaches for Assessing Atmospheric Deposition of Potentially Toxic Elements

**DOI:** 10.3390/molecules31132393

**Published:** 2026-07-07

**Authors:** Paweł Świsłowski, Małgorzata Rajfur, Tymoteusz Turlej, Inga Zinicovscaia, Oznur Isinkaralar, Kaan Isinkaralar, Anca-Iulia Stoica

**Affiliations:** 1 Institute of Biology, University of Opole, 45-032 Opole, Poland; rajfur@uni.opole.pl; 2 Department of Power Systems and Environmental Protection Facilities, AGH University of Krakow, 30-059 Cracow, Poland; turlej@agh.edu.pl; 3 Horia Hulubei National Institute for R&D in Physics and Nuclear Engineering, 077125 Magurele, Romania; zinikovskaia@mail.ru; 4 Department of Landscape Architecture, Faculty of Engineering and Architecture, Kastamonu University, Kastamonu 37150, Türkiye; obulan@kastamonu.edu.tr; 5 Department of Environmental Engineering, Faculty of Engineering and Architecture, Kastamonu University, Kastamonu 37150, Türkiye; kisinkaralar@kastamonu.edu.tr; 6 Institute of Sanitary Engineering and Water Pollution Control (SIG), University of Natural Resources and Life Sciences, 1190 Vienna, Austria; anca.stoica@boku.ac.at

**Keywords:** atmospheric deposition, moss-bag biomonitoring, PM_10_, potentially toxic elements, relative accumulation factor

## Abstract

PM_10_ filters provide short-term quantitative information on particle-bound potentially toxic elements (PTEs), whereas mosses integrate deposition and accumulation over longer periods but do not provide air-volume-normalised concentrations. Their combined use may therefore provide a more complete assessment of atmospheric PTE deposition. The study aimed to assess whether active moss biomonitoring and filter-based PM_10_ monitoring provide complementary information on atmospheric deposition of PTEs under comparable exposure conditions. During the six-month campaign in Opole, PM_10_ was collected during repeated 24 h sampling events, while three moss species: *Pleurozium schreberi*, *Sphagnum fallax*, and *Dicranum polysetum* were exposed cumulatively. PTE concentrations were determined by ICP-MS; particle-size descriptors, including Q_10_, Q_50_, and Q_90_, were analysed for a subset of filters, whereas net concentration change and *RAF* were calculated relative to identically processed unexposed moss controls. Spearman correlation, PCA, and Bray–Curtis dissimilarity were used for data analysis. The material retained on the PM_10_ filters was dominated by Fe, Zn, and Pb, whilst elevated peak values for Cd, Zn, and Pb indicated episodic enrichment in some samples. In mosses, Pb and Co showed the most consistent relative enrichment, while mean *RAF* exceeded 1.0 for five elements in *P. schreberi* and two elements each in *D. polysetum* and *S. fallax*. PCA separated PM_10_ from moss profiles, with the first two components explaining 80.4% of the variance, while PM_10_-moss Bray–Curtis distances ranged from 0.75 to 0.81. The results indicate that PM_10_ filters and mosses record different but complementary aspects of the atmospheric PTE signal. The simultaneous use of both methods allows the atmospheric PTE signal to be interpreted at two levels: the short-term composition of PM_10_ material retained on the filters, and the long-term retention and accumulation of elements within the moss matrix.

## 1. Introduction

Potentially toxic elements (PTEs) are a significant component of atmospheric pollution, as they are found primarily in particulate matter in urban and industrial air. Their environmental significance stems not only from the toxicity of selected elements, such as Cr, Ni, As, Cd or Pb, but also from their persistence, variable mobility and dependence on the properties of the particles to which they are bound. Contemporary research on PTEs in atmospheric dust increasingly goes beyond the mere determination of total concentrations, as the form of element binding, their distribution across particle fractions, and potential bioavailability influence the assessment of environmental risk and the interpretation of emission sources [1]. In urban environments, the PTE profile in PM may reflect a mixture of combustion emissions, resuspension of road dust, and wear from tyres, brakes and road surfaces; moreover, the contribution of non-combustion emissions to traffic-related pollution is now increasingly highlighted as a significant component of particulate matter exposure [2,3,4].

PM_10_ filter monitoring provides an instrumental and quantitative record of the composition of particulate matter captured during a specific sampling interval. Given the known air volume passed through and the mass of deposited dust, it is possible to link PTE concentrations to a specific filter sample and a defined exposure time. This is the fundamental advantage of filters: the result is technically well-defined and relates to the material captured by the device under controlled conditions. At the same time, a PM_10_ filter does not directly show long-term retention or biological accumulation of pollutants. It records the composition of the particle fraction at the time of sampling but does not indicate the extent to which PTEs are retained on biological surfaces or how their signals integrate over a longer period [1,5].

Active biomonitoring using mosses provides a different type of information. Mosses placed in bags do not act as a filtration device or measure air quality in real time. They serve as a biological matrix that integrates the deposition, retention and accumulation of PTEs during the exposure period. Their usefulness stems from their large surface area exposed to the atmosphere, high sorption capacity, and limited substrate influence on element uptake. Still, the results depend on the species, initial concentration, exposure time, and properties of the retained particles. Therefore, in active biomonitoring studies, analysis of final concentrations alone is insufficient. It is necessary to account for the net increase and the relative accumulation factor (*RAF*), which helps mitigate the impact of initial differences between moss species [6,7,8].

The two approaches differ in temporal resolution and retention mechanisms. PM_10_ filters collect a size-defined particulate fraction during discrete sampling intervals, whereas mosses integrate deposition over longer periods through particle interception, surface sorption, and cation exchange [5,9]. Moss accumulation may therefore depend on particle size, the chemical form and binding affinity of individual elements, species-specific surface properties, rainfall-related leaching or wash-off, and wind-dependent particle interception and retention [10,11]. Consequently, filter and moss profiles should not be expected to correspond directly, even when both reflect contributions from the same atmospheric sources.

Comparing mosses and filters only makes sense if both methods are treated as complementary rather than interchangeable. A previous study on the active biomonitoring of PTEs and TSP filters showed that the results of the two approaches are not fully consistent, as mosses and filters represent different matrices, mechanisms of pollutant retention and timescales [5]. A similar principle of complementarity was applied in a comparative study of PAHs, in which filters better reflected the short-term signal of particulate matter, whilst mosses recorded the cumulative accumulation of compounds over the duration of exposure [12]. However, these studies left two specific PTE-related gaps. The TSP-based comparison did not isolate the PM_10_ fraction or examine element-specific relationships with particle-size descriptors, whereas the PAH study addressed a different pollutant class and therefore did not resolve PTE-specific accumulation expressed as ΔC and *RAF*. The present study addresses these gaps by linking PM_10_-bound PTE concentrations and deposited dust mass with particle-size descriptors and species-specific moss accumulation during a six-month campaign. The same three moss species were retained to support comparability with the previous studies.

The mass of dust captured on the filter and the particle size characteristics provide relevant physical context for interpreting this comparison. The mass of deposited material determines the actual dust load on the sample, whilst descriptors such as CE Diameter, Q_10_, Q_50_, Q_90_, the mean, and standard deviation describe the particle-size distribution of particles captured on the filters. Particle size can influence atmospheric transport, deposition and the transport capacity of PTEs, but it is not the only factor explaining the chemical composition of dust. The relationship between particles and accumulation in mosses may also depend on emission sources, resuspension, mineral composition, local conditions and the surface characteristics of the gametophyte. The importance of this approach is confirmed by studies in which the quantity and nature of particles captured by moss-bags were linked to the elemental composition of the biomonitors [13]. In this study, particle-size descriptors are therefore used as contextual physical information to support the interpretation of PM_10_-bound PTE profiles, rather than as a primary explanatory factor or a direct predictor of moss accumulation.

To address these gaps, this study tested whether combining PM_10_-bound PTE concentrations and particle-size descriptors with cumulative, species-specific moss accumulation within the same six-month field campaign provides complementary information that neither approach can deliver independently. The proposed analytical framework linked three levels of evidence: (i) the chemical composition of PM_10_ material retained on quartz filters, (ii) the mass and particle-size characteristics of the retained particulate matter, and (iii) species-specific changes in PTE concentrations in *Pleurozium schreberi*, *Sphagnum fallax*, and *Dicranum polysetum*. Changes in moss concentrations were expressed as net concentration change (ΔC) and the Relative Accumulation Factor (*RAF*). We hypothesised that PM_10_ filters and mosses would not produce convergent elemental profiles because they differ in matrix properties, retention mechanisms, and temporal integration, but that the information obtained from both approaches would be complementary. Accordingly, we expected particle-size descriptors to show element-specific rather than uniform relationships with PM_10_-bound PTEs, the three moss species to exhibit different accumulation patterns, and multivariate analyses to distinguish PM_10_ and moss profiles while identifying similarities among the moss species. This framework allowed both approaches to be evaluated as complementary rather than interchangeable tools for atmospheric PTE assessment.

## 2. Results

### 2.1. The Elemental Composition of PM_10_ Material Captured on Filters and Its Relationship with Particle Size Descriptors

The concentrations of PTEs in PM_10_ collected on the filters showed marked variation among elements (Table 1). The highest median values were recorded for Fe, Zn, and Pb at 10,621, 1739, and 543 µg/g, respectively. Lower but still significant median values were observed for Cu, Mn, and Ba at 269, 241, and 130 µg/g, respectively. The lowest median values were found for Co, V, and Cd at 5.99, 16.2, and 16.4 µg/g, respectively. The filtration profile was therefore dominated by Fe, Zn and Pb, whilst Co, V and Cd occurred at lower levels.

The ranges of values indicated considerable variability in the composition of PM_10_ material among filter samples. The largest maximum mass fractions in the PM_10_ material were recorded for Fe, Zn, Cd, and Pb, at 40,847, 22,184, 9764, and 4306 µg/g, respectively. These values describe the elemental composition of the collected particulate material and should not be interpreted directly as ambient-air concentrations or regulatory exceedances. The particularly large difference between the median and the maximum for Cd indicates episodic increases in this element in some of the samples. A similar, though less extreme, pattern was observed for Zn and Pb. For this reason, medians and ranges of values better characterise the typical filtration profile than arithmetic means alone.

**Figure 1:**
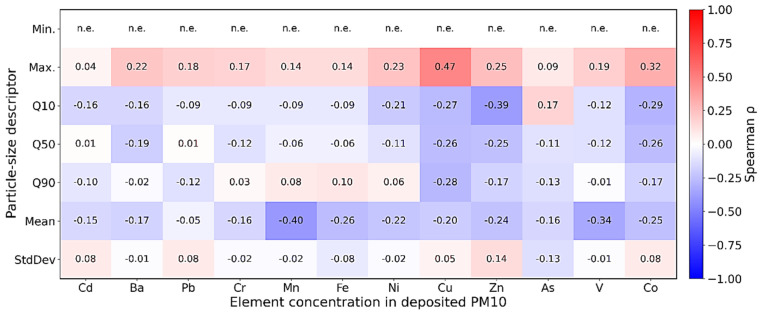
Spearman correlations between particle-size descriptors and PM_10_-bound PTE concentrations. Positive coefficients indicate that higher descriptor values were associated with higher PTE concentrations, whereas negative coefficients indicate inverse relationships; increasing colour intensity represents increasing correlation strength. n.e., not estimable; Min. was constant across all analysed samples (0.16 µm), and therefore Spearman’s ρ could not be calculated.

The particle size descriptors of the material deposited on the filters are presented in the Supplementary Materials (Table S1 SM). The minimum value of the particle size descriptor was constant across all analysed samples and amounted to 0.16 µm; therefore, Spearman’s correlation coefficient for this parameter could not be estimated and is marked as ‘n.e.’ in Figure 1. This notation does not indicate a lack of data, but rather a lack of variability in the input variable.

Spearman’s correlations between particle size descriptors and PTE concentrations in the PM_10_ material were selective and element-specific (Figure 1). The strongest positive correlation was found between the Max. value and Cu (ρ = 0.47). Weaker positive correlations were also observed for Max. with respect to Co, Zn, Ni and Ba, with ρ = 0.32, 0.25, 0.23 and 0.22, respectively. The highest negative correlations were observed for the relationships between Mean and Mn, Q_10_ and Zn, and Mean and V, with ρ values of −0.40, −0.39, and −0.34, respectively. Negative correlations were also observed for Q_10_ and Co, Q_90_ and Cu, Q_10_ and Cu, and Q_50_ and Cu.

Overall, the correlations were weak to moderate and element-specific, indicating that no uniform relationship existed between particle-size descriptors and PM_10_-bound PTE concentrations. Because these relationships did not show a consistent element-wide pattern, particle-size descriptors were treated as contextual information rather than as direct predictors of moss accumulation. The subsequent *RAF* analysis therefore examines cumulative and species-specific element accumulation in mosses as a complementary line of evidence, rather than as a direct continuation of the particle-size analysis.

### 2.2. The Accumulative Response of Mosses Based on RAF

**Figure 2:**
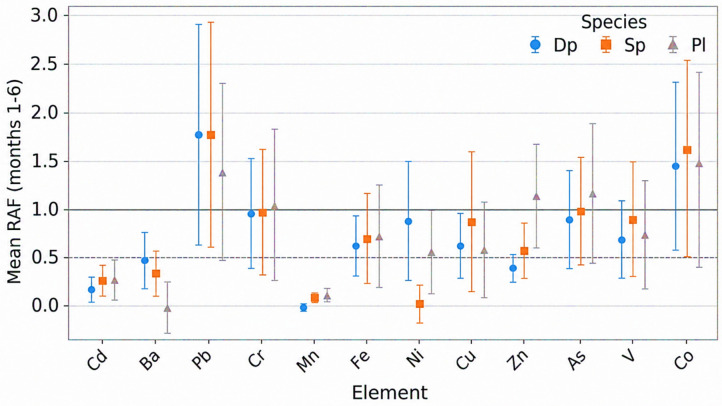
Mean *RAF* values for three moss species across six cumulative exposure endpoints (months 1–6). Markers show the mean of the six endpoint-specific values, and error bars represent the standard deviation among these values. The solid and dashed horizontal lines indicate *RAF* values of 1.0 and 0.5, respectively.

The *RAF* values indicated element- and species-specific accumulation responses (Figure 2). The graph shows the mean *RAF* values calculated from six months of exposure, and the error bars represent the standard deviation among the six endpoint-specific means. Higher *RAF* values represent stronger enrichment relative to the initial moss material, while the horizontal lines at 0.5 and 1.0 indicate the thresholds used to support interpretation. The highest average *RAF* values were obtained for Pb and Co. For Pb, the average *RAF* was 1.78 in *D. polysetum*, 1.77 in *S. fallax* and 1.38 in *P. schreberi*. For Co, these values were 1.45, 1.62 and 1.48, respectively. These two elements showed the most consistent relative enrichment in the moss species studied.

Mean *RAF* exceeded 1.0 for five elements in *P. schreberi* (Pb, Cr, Zn, As, and Co) and for two elements in both *D. polysetum* and *S. fallax* (Pb and Co). Additional elements showed slight enrichment within the *RAF* range of 0.5–1.0, whereas Mn, Cd, and Ba generally remained below 0.5 (Figure 2). Despite these differences in element-specific profiles, the mean *RAF* calculated across all analysed elements was nearly identical among species: 0.74 for *D. polysetum*, 0.75 for *S. fallax*, and 0.76 for *P. schreberi*. Thus, the nearly identical overall mean *RAF* values do not indicate substantial differences among the three moss species in overall relative accumulation capacity. The descriptive interspecific variation concerned mainly the composition of the enrichment profile, i.e., which elements exceeded the *RAF* thresholds, rather than the overall magnitude of accumulation.

### 2.3. Internal Ranking of Moss Accumulation Signals

The internal accumulation-based ranking, calculated from net PTE accumulation in mosses, was used to rank the relative contribution of elements to the accumulation signal (Table 2). These results do not constitute a conventional assessment of ecological risk across the entire study area, but rather a ranking of contamination in the moss material, based on concentration changes during exposure.

The highest index value was recorded for sample Pl4, with a contribution score of 212. The main contributor was Zn, followed by Pb. High values were also recorded for Pl6 and Pl5 (156 each), as well as for Sp6, Sp5, Pl3, Dp4, Dp6 and Dp5. The highest-ranking values, therefore, occurred mainly in the later months of exposure but were not limited to a single species.

The total contribution of species to the overall accumulation-based score was highest for *P. schreberi*, which accounted for 733 units, or 42.6% of the total index value. The contribution of *D. polysetum* was 497 units, or 28.9%, whilst that of *S. fallax* was 490 units, or 28.5%. This pattern is consistent with the *RAF* results, in which *P. schreberi* showed the highest number of elements exceeding the threshold for clear relative enrichment.

Pb, Zn and Cu made the largest contribution to the total accumulation-based score. Their contributions were 572, 534, and 451 units, respectively, corresponding to 33.3%, 31.1%, and 26.2% of the total index value. The remaining elements had a markedly smaller share: Ni 3.4%, As 2.2%, Cd 2.2% and Cr 1.7%. The ranking, therefore, orders the elements according to their relative contribution to the moss accumulation signal, but does not replace a direct interpretation of net accumulation or a comparison of PM_10_-moss profiles.

### 2.4. A Multidimensional Comparison of PM_10_ Profiles and Mosses

**Figure 3:**
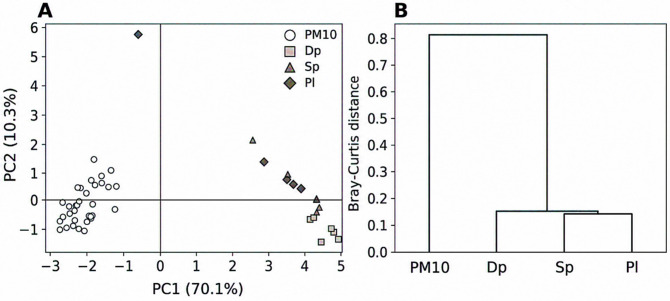
Multivariate comparison of PM10 filter and moss PTE profiles. (**A**) PCA scores plot; separation along PC1 indicates the main compositional difference between PM_10_ and moss profiles. (**B**) Bray–Curtis dendrogram of normalised PTE profiles; shorter linkage distances indicate greater similarity. The three moss species formed a closely related group that was clearly separated from the PM_10_ profile.

A multidimensional comparison of PTE profiles revealed a clear separation between PM_10_ samples and moss profiles (Figure 3). The first two PCA components together explained 80.4% of the data variability, with PC1 accounting for 70.1% and PC2 for 10.3%. The PM_10_ samples were located on the negative side of PC1, whilst most of the moss profiles were distributed on the positive side of this axis. The main gradient of variation thus separated the filter material from the biomonitoring profiles.

Descriptive differences among moss species were observed within the moss profiles. Samples of *D. polysetum* formed a relatively compact cluster on the positive side of PC1 and the negative side of PC2. Samples of *S. fallax* were also located on the positive side of PC1, but were shifted further towards positive PC2 values. Most *P. schreberi* samples occupied positions similar to the other moss profiles, whereas Pl1 was clearly shifted relative to the other samples of this species. This indicates a different profile in the first month of exposure, whilst maintaining the overall separation of moss profiles from PM_10_.

The Bray–Curtis dendrogram confirmed the separation of the filter profile from the moss profiles. The Bray–Curtis distances between PM_10_ and the moss profiles were high, with values of 0.81 for PM_10_-Dp, 0.76 for PM_10_-Sp, and 0.75 for PM_10_-Pl. The *P. schreberi* profile showed the smallest distance relative to PM_10_, but the similarity remained low. By comparison, the distances between the moss profiles were substantially lower: 0.14 for Sp-Pl, 0.15 for Dp-Sp and 0.15 for Dp-Pl. The profiles of the three moss species were therefore more similar to one another than to the PM_10_ profile. Taken together, both analyses indicate that the principal difference occurred between the monitoring matrices, whereas the elemental profiles of the three moss species were comparatively similar.

The divergence between PM_10_ profiles and moss data does not imply inconsistencies in the methods. This result reflects the differences between the short-term record of particulate matter composition on filters and the integrated, biologically modulated accumulation in mosses. PM_10_ filters capture the elemental composition of particles collected during specific sampling intervals, whereas mosses integrate the deposition, retention, and accumulation of PTEs over the duration of exposure. The observed separation of profiles indicates that active biomonitoring using mosses and filter-based monitoring provides complementary rather than interchangeable information.

## 3. Discussion

### 3.1. Complementarity of PM_10_ Filter Monitoring and Active Moss Biomonitoring

PM_10_ filter monitoring and active moss biomonitoring captured different but complementary aspects of the atmospheric PTE signal. Filters characterised the elemental composition of particulate matter collected during discrete sampling intervals, whereas mosses integrated deposition, retention, and accumulation over the exposure period. Therefore, the lack of full agreement between their elemental profiles reflects differences in matrices, retention mechanisms, and temporal resolution rather than methodological inconsistency. This qualitative matrix separation is consistent with earlier comparisons involving TSP-bound PTEs and PAHs, in which filter-based and moss-based profiles also represented distinct analytical signals [5,12].

PCA and Bray–Curtis analyses confirmed a clear separation between PM_10_ and moss profiles, while the three moss species were substantially more similar to one another than to the filter material. This indicates that mosses do not directly reproduce the elemental composition of PM_10_ but transform the deposition signal through species-specific surface and biological properties. Similar caution in comparing moss species and analytical approaches has been emphasised in previous active biomonitoring studies [6,8].

### 3.2. PM_10_-Bound PTE Profiles and Particle-Size Relationships

The PTE profile in the PM_10_ material was dominated by Fe, Zn, and Pb, indicating the presence of a mixture of urban particle sources. These potentially include the resuspension of mineral material, traffic emissions, and wear from tyres, brakes, and road surfaces, as well as local industrial and municipal sources. However, this interpretation should be treated as a probable one rather than a formal identification of sources, as no classical source apportionment analysis was performed in the study. Recent reviews on PTEs associated with atmospheric particulate matter emphasise that the chemical composition of PM depends simultaneously on emission sources, particle fractions, the chemical forms of elements and local conditions [1]. Reviews and recent studies on non-exhaust emissions indicate that tyre wear, brake wear, road surface wear and the resuspension of road dust can significantly contribute to the composition of PM in urban environments, including its metallic fraction and particle size distribution [2,3,4,14].

The large discrepancy between the median and maximum values for selected elements, particularly Cd, Zn and Pb, suggests that the chemistry of PM_10_ was not determined solely by stable background levels. Part of the filter profile may have reflected episodic inflows of material with elevated levels of specific PTEs. This is consistent with the heterogeneous nature of urban dust, whose composition may vary with local emissions, resuspension, meteorological conditions and short-term pollution episodes [1,15].

The values reported in Table 1 represent elemental mass fractions in the collected PM10 material (µg/g), rather than ambient-air concentrations (ng/m^3^), and therefore cannot be directly compared with regulatory air-quality limits. In the broad urban-dust compilation of Kaonga et al. [15], the median Fe, Zn and Pb contents observed in this study were within or comparable to values reported for urban environments, whereas the maximum Zn and Pb contents indicated marked enrichment. The maximum Cd value was exceptionally high relative to published ranges and should be interpreted as an isolated extreme. These comparisons are only contextual because urban dust and filter-collected PM_10_ are not equivalent matrices. Consequently, the results indicate compositional enrichment but do not demonstrate regulatory exceedance or quantify health risk, which would require air-volume-normalised concentrations, exposure data, chemical speciation and bioaccessibility [15,16].

The relationships between particle size descriptors and PTE concentrations were selective and element-specific. The clearest positive correlation between the maximum particle size and Cu suggests that some of the Cu may have been associated with larger suspended particles or with episodes of coarser deposition. Conversely, the negative relationships for Mean-Mn, Q_10_-Zn and Mean-V indicate that, for some elements, the finer fraction of the particle size distribution or source variability not linearly related to a simple increase in particle size may have been more significant. The literature emphasises that metals associated with PM may exhibit particle-size dependence. Still, the direction and strength of this relationship depend on the source, mineral composition and chemical form of the element [1].

However, these relationships should be treated as a limiting result rather than overinterpreted. The correlations were weak to moderate and did not yield a single universal pattern across all PTEs (Figure 1), indicating that particle-size descriptors did not provide a general explanatory variable for the elemental composition of the collected PM_10_ material. This affected the interpretation of the study design: particle-size descriptors were retained as contextual physical information, whereas the filter–moss comparison was based primarily on elemental profiles, *RAF* patterns, and multivariate dissimilarity. Particle size may influence atmospheric transport, deposition, sorption surface area, and potential metal transport capacity, but it cannot replace information on emission sources, mineral composition, particle morphology, resuspension, chemical speciation, or local conditions. Morphological and mineralogical studies of PM across different size fractions show that composition and origin of particles can vary significantly among PM_10_, PM_2.5_, and PM_1_, which limits the ability to interpret particle size alone as a single predictor of chemical composition [17].

It is also important to note that the descriptors refer to the numerical distribution of particles captured on the filters, rather than the total mass fraction of PM_1_, PM_2.5_, or PM_10_. For this reason, the interpretation should remain cautious: the results indicate that particle size partly accounts for the chemical variability of PM_10_ but does not unequivocally determine it.

### 3.3. Species-Specific Moss Accumulation and RAF Interpretation

The *RAF* values indicated descriptive accumulation patterns that varied by element and moss species. The most consistent relative enrichment was observed for Pb and Co, which exceeded the *RAF* threshold of >1.0 in more than one species. Descriptively, *P. schreberi* showed the widest range of elements with *RAF* > 1.0, including Pb, Cr, Zn, As, and Co, whereas *D. polysetum* and *S. fallax* exceeded this threshold mainly for Pb and Co (Figure 2). This descriptive pattern suggests that the three moss species did not differ substantially in overall relative accumulation capacity, but differed mainly in the profile of elements undergoing enrichment.

The mean *RAF* values obtained for Pb (1.38–1.78) and Co (1.45–1.62) indicate considerable relative enrichment according to the commonly applied *RAF* classification. These values correspond to exposed-moss concentrations approximately 2.38–2.78 times the initial concentration for Pb and 2.45–2.62 times the initial concentration for Co. For comparison, lower mean *RAF* values for Pb (0.31–0.67) were reported in a six-week urban traffic study using *Sphagnum junghuhnianum*, whereas high *RAF* values for Co have also been observed in urban moss-bag monitoring [6,18]. However, such comparisons remain contextual because *RAF* depends on moss species, initial element content, exposure duration, meteorological conditions, and bag preparation. Pb is of particular public-health relevance because chronic exposure, including at low levels, is associated with neurological and cardiovascular effects, while excessive exposure to Co may also produce adverse systemic effects [19,20]. Nevertheless, *RAF* does not quantify atmospheric deposition flux, ambient-air concentration, inhaled dose, chemical speciation, or bioaccessibility. Therefore, the observed values demonstrate a clear accumulation signal for Pb and Co but do not, by themselves, establish a human-health hazard. *RAF* and ΔC were therefore interpreted as complementary descriptors of relative and net accumulation, rather than combined into a single weighted enrichment index requiring additional assumptions and validation.

At the same time, the mean *RAF* calculated across all elements was very similar among the species. This result is important from an interpretative perspective, as it does not allow for a straightforward identification of a single “best” biomonitoring species in terms of total accumulation. It is more justified to state that the individual species differed in the range and structure of their elemental responses. *P. schreberi* showed a broader enrichment profile, whereas *D. polysetum* and *S. fallax* exhibited a more selective response for selected elements. A similar distinction between the overall effectiveness of a biomonitor and an element-specific accumulation pattern has been reported in active urban biomonitoring studies, where *RAF* and other enrichment indices were interpreted as measures of relative response to exposure rather than as direct equivalents of airborne concentrations [6,8,21].

Differences among species may result from morphological and physicochemical characteristics of mosses, such as gametophyte architecture, the surface area exposed to aerosols, particle retention capacity, hygroscopicity, leaf structure, and initial concentration. Moss-bag studies show that the amount and nature of particles captured by mosses may be associated with elemental accumulation, but these relationships are modulated by land use, emission sources, and the properties of the biological matrix itself [13,22].

This is why the interpretation of active biomonitoring results should be based on net increase and RAF rather than solely on final concentrations. The concentration measured after exposure may reflect both actual accumulation during the campaign and initial differences among species. *RAF* helps reduce this problem by relating the change in concentration to the initial level of a given species. More recent studies on moss transplants have emphasised that indices based on enrichment relative to the initial material are useful but also sensitive to the quality and variability of the initial controls; therefore, their interpretation requires the assumptions and limitations to be explicitly stated [8].

Values below the enrichment thresholds, particularly for Mn, Cd, and Ba, indicate that not all PTEs were effectively retained or accumulated by the mosses in the studied system. This may result from reduced availability of these elements during deposition, differences in their particle-bound forms, possible leaching, or limited retention on the gametophyte surface. Therefore, the absence of an increase in *RAF* for a given element does not automatically indicate its absence in PM_10_, but rather the lack of clear relative enrichment in the mosses.

### 3.4. Interpretation of the Internal Accumulation-Based Ranking

The risk-related ranking based on net PTE accumulation in mosses provided a synthetic ordering of elements according to their contribution to the accumulation signal. Accordingly, the ranking should be interpreted only as a relative prioritisation of accumulation signals within the analysed moss dataset, not as a quantitative estimate of ecological risk. Pb, Zn, and Cu contributed the most to the total score, indicating that these elements were key drivers of moss loading during exposure (Table 2). At the species level, *P. schreberi* showed the highest contribution, which is consistent with the descriptive *RAF* pattern showing the largest number of elements above the threshold for clear relative enrichment.

Among the analysed elements, Pb provided the clearest concordant signal across both monitoring approaches. Its median and maximum mass fractions in the PM10 material were 543 and 4306 µg/g, respectively, while mean *RAF* values ranged from 1.38 to 1.78 in all three moss species; Pb also contributed 33.3% to the accumulation-based score. Although these metrics are not directly comparable, their convergence identifies Pb as a priority element for further environmental and exposure-oriented monitoring. In contrast to Fe and Zn, which are essential micronutrients but may become toxic in excess, Pb and Cd have no known physiological function in mosses [23]. The elemental burden measured in moss may include surface binding and retained particles, rather than only intracellular uptake [13]. Growth, chlorophyll fluorescence, oxidative stress, membrane integrity and viability were not assessed, the present study cannot determine whether Pb or other PTEs caused sublethal toxicity or reached lethal levels in the mosses [23].

This part of the results, however, should be interpreted with caution. The indices applied here do not constitute a classical ecological risk assessment for the entire study area, as they do not refer directly to soil, sediments, or the full atmospheric deposition flux. Classical ecological risk indices were originally developed mainly for other environmental matrices, especially sediments; therefore, their direct transfer to moss material may lead to overinterpretation [24]. In the present study, these indices were used solely for the relative ranking of elements net-accumulated in mosses, and not for a classical ecological risk assessment of the entire study area. This limitation is consistent with more recent approaches, which recommend careful selection of indices for moss transplants and a clear distinction between accumulation assessment and formal environmental risk assessment [8].

### 3.5. Limitations and Implications for Future Monitoring

The main limitation of this study is the difference in matrices, units, and temporal resolution. PM_10_ filters characterised particulate material collected during discrete sampling intervals, whereas mosses integrated retention and accumulation over a longer exposure period. Consequently, direct numerical comparison between the two datasets is not appropriate [5,12].

A second limitation is the nature of the particle-size analysis. Particle descriptors provide important physical context, but they do not replace a full analysis of PM mass fractions or the chemical speciation of PTEs. The lack of information on the chemical forms of the elements limits the interpretation of bioavailability and binding mechanisms on the moss surface. In studies of PTEs associated with atmospheric particles, the total elemental content alone is not sufficient for a full assessment of mobility, availability, and potential risk, as chemical forms and operational fractions are of major importance [1].

A third limitation is the selectivity of the indices used. Enrichment Factor (*EF*) was not employed as the primary interpretative index because the classical application of EF requires a consistently justified reference element and geochemical background. In the present study design, ΔC, *RAF*, and the relative risk-related ranking based on net accumulation were more robust from an interpretative perspective. These indices enable the structuring of the accumulation signal in moss material, but they should not be treated as a comprehensive ecological risk assessment of the study area.

Future studies should include greater biological replication, meteorological data, PTE speciation, direct assessment of particle retention on the gametophyte surface, and formal source-apportionment approaches, including PMF [25].

## 4. Materials and Methods

### 4.1. Study Area and Experimental Design

The study was conducted in Opole, in south-western Poland, under urban atmospheric exposure conditions. The PM_10_ sampler and moss bags were colocated at the same sampling station on an elevated, unobstructed second-floor outdoor viewing platform, at the same platform level and in close proximity to one another. No walls, vegetation, or other immediate obstacles separated the two monitoring systems, providing both with comparable exposure to the same local air mass. The campaign lasted six months, from 21 August 2021 to 21 February 2022, covering late summer, autumn, and winter conditions. Monthly air-temperature data (minimum, mean, and maximum) and solar radiation were available for the same six-month campaign and were previously analysed in the parallel PAH study [12]. It involved the parallel application of two approaches to assessing PTE deposition: active biomonitoring using moss bags and instrumental monitoring of PM_10_ on quartz filters. This experimental setup enabled a comparison between the long-term, biologically integrated accumulation signal in mosses and the short-term record of particulate matter composition retained on PM_10_ filters. The study period spanned the transition from late summer through autumn to winter, which was significant for interpreting deposition variability, as emission sources, dust dispersion conditions and the intensity of heating emissions may change during this time [26].

The study design was based on a direct comparison of biological and filtration exposure, rather than on a comparison of data from different campaigns. This is important because mosses and filters capture different aspects of the atmospheric pollution signal. PM_10_ filters document the composition of particles captured during a specific sampling period, whereas mosses integrate the retention and accumulation of PTEs over successive months of exposure. A similar field setup was used in a six-month campaign on PAHs; however, the present study focuses on PTEs and their relationships with dust mass and particle-size descriptors [12].

### 4.2. Moss Material and Active Biomonitoring

Three species of moss were used for active biomonitoring: *Pleurozium schreberi* (Pl), *Sphagnum fallax* (Sp) and *Dicranum polysetum* (Dp). The species were selected because all three had previously been used in active moss-bag biomonitoring and in comparative filter–moss studies, which supported methodological continuity and cross-study comparability [5,11,12]. Their joint use also enabled the assessment of interspecific variation in PTE accumulation, as moss species may differ in particle retention and element-binding capacity [11]. All moss material was collected from the same background area to minimise differences in initial exposure history. The moss material was collected from a forest area that serves as the environmental background in the Świętokrzyskie Province. Before exposure, the material was prepared in accordance with the principles of active biomonitoring using bryophytes and the recommendations applied in European biomonitoring studies [27,28]. Visible plant debris and soil particles were removed manually. The pooled moss material was conditioned in demineralised water according to the procedure [28]. After conditioning, the material was dried at room temperature before being placed in the moss bags.

Two grams of fresh moss material were placed in each moss bag. For each species and each scheduled exposure endpoint, two moss bags were exposed in parallel. The experimental design therefore comprised 36 exposed bags: three species × six cumulative exposure periods × two parallel exposure replicates. Both bags assigned to a given endpoint were collected after 1, 2, 3, 4, 5, or 6 months, and their results were averaged to represent the corresponding species × exposure-time combination. One unexposed initial sample of each species was used as the reference for the calculation of ΔC and *RAF*. Because all bags were deployed at the beginning of the campaign and collected at successive endpoints, the samples represented cumulative exposure periods rather than independent one-month exposures. Each moss sample was digested in triplicate as technical replication.

### 4.3. PM_10_ Filter Sampling and Particulate Load Calculation

In parallel with the moss exposure, PM_10_ sampling was carried out using 47 mm diameter QM-A quartz filters (Whatman, Maidstone, UK). A PNS3D15/LVS3D particulate matter monitor (ATMOSERVICE Sp. z o.o., Poznań, Poland) equipped with a PM_10_ head was used for the sampling. The air flow rate was 2.3 m^3^/h, and the sampling time for a single filter sample was 24 h. Each filter represented a discrete 24 h sampling interval within the six-month campaign and was replaced after each sampling event. Sampling was conducted on selected dates rather than continuously throughout the campaign. Elemental concentrations were determined in the collected PM_10_ material, while the correlation analysis included only filters for which both PTE concentrations and particle-size descriptors were available. Filter samples were not matched one-to-one with individual moss exposure endpoints. The same instrument type and flow rate were used in a previous study comparing active biomonitoring with mosses to TSP collection on filters; however, in the current campaign, a PM_10_ head was used, as in the six-month PAH campaign [5,12]. This distinction is important because PM_10_ and TSP do not describe the same fraction of particulate matter, and mosses can trap particles of varying sizes.

For each filter, the mass of the filter with the deposited material, the mass of the filter alone, and the mass of the deposited dust sample were determined. The concentrations of PTEs in the filters were expressed as µg/g relative to the mass of the deposited dust sample. The filter results were expressed as elemental mass fractions in the collected PM_10_ material because the study focused on comparing elemental profiles between filters and mosses rather than on assessing air-volume-normalised concentrations or compliance with air-quality standards. This means that the primary unit of interpretation for the filters is the chemical composition of the PM_10_ material retained on the filter, rather than the mass of the entire filter.

For a subset of the 47 mm quartz filters, particle-size analysis was performed using a Morphologi 4 automated particle imaging system (Malvern Panalytical, Malvern, UK), following the imaging procedure described previously [12]. Each filter was mounted in a dedicated 47 mm filter holder, and images were acquired from multiple 4 mm^2^ measurement fields distributed across the filter surface. Imaging was performed using a 50× objective under episcopic bright-field illumination with Z-stacking, and particles were identified using the Low Contrast segmentation algorithm.

The particle-size distribution was evaluated on a number basis using the circle-equivalent diameter (CE Diameter), defined as the diameter of a circle with the same projected area as the detected particle. The software-derived Q_10_, Q_50_, and Q_90_ values represent the CE Diameters below which 10%, 50%, and 90% of the detected particles occurred, respectively. Minimum, maximum, mean, and standard-deviation values of CE Diameter were also obtained for each analysed filter.

These variables describe the numerical characteristics of the particles retained on the filters and were used as a mechanistic context for interpreting the chemical composition of PM_10_. The particle data were linked to PTE measurements in the same or corresponding filter samples, covering, among others, Cd, Ba, Pb, Cr, Mn, Fe, Ni, Cu, Zn, As, V, Co and Hg.

### 4.4. Sample Preparation, Digestion, ICP-MS Analysis and Quality Control

After exposure, the moss samples were not washed to avoid removing particles retained during the field campaign. Moss and filter samples were dried at room temperature until constant mass was achieved. The moss material was ground in an agate mortar, passed through a 100-mesh sieve, and homogenised in a mixer for 10 min. Precisely weighed portions of 0.1 g of dry moss were used for digestion. For the filter samples, the entire dried 47 mm quartz membrane, with a mass of approximately 0.15 g, was transferred to the digestion vessel. Moss and filter samples were microwave-digested in an MLS Mega 1200 system (Milestone, Sorisole, Italy) using an HNO_3_/H_2_O_2_ mixture in accordance with EN ISO 15587-2:2002 [29]. After digestion, the solutions were filtered to remove residual insoluble material and diluted with ultrapure water to the final analytical volume before ICP-MS analysis. The unexposed initial moss samples were dried, ground, homogenised, digested, and analysed using the same procedures as the exposed moss samples.

Element concentrations were determined by ICP-MS using a PerkinElmer^®^ NexION^®^ 2000 Inductively Coupled Plasma Mass Spectrometer (ICP-MS; PerkinElmer Inc., Waltham, MA, USA), in accordance with DIN EN ISO 17294-2 (E 29):2004, which relates to the determination of elements by ICP-MS [30]. The main ICP-MS operating and data-acquisition parameters are provided in Supplementary Table S2. An eight-point calibration curve was prepared for each element analysed, and the linear correlation coefficient for all calibration curves was greater than 0.999. Each moss sample was digested three times to verify the repeatability of the sample preparation process.

The accuracy and reproducibility of the analytical results were monitored at several levels. The laboratory participated in annual proficiency testing and interlaboratory comparison exercises. A laboratory performance check was conducted daily before analysis using a certified PerkinElmer solution to verify detector performance, plasma conditions, and signal stability. Blank and control samples were analysed at the beginning and end of each measurement sequence, and their results were evaluated according to the acceptance criteria specified in ISO/TS 13530:2009 [31].

### 4.5. Data Treatment: Net Accumulation, RAF, Accumulation-Based Contribution Ranking and Moss-Filter Harmonisation

Data for mosses were analysed separately for each species and each month of exposure. As the three species may have differed in their initial PTE content, the primary interpretative variable was net growth [22], calculated as:

$$\Delta C=C_{exposed}-C_{initial}$$

where $C_{exposed}$ denotes the concentration of a given element in a moss sample after a specified exposure time, and $C_{initial}$ denotes the concentration of that element in an initial sample of the same species. The initial values corresponded to the control samples. This approach makes it possible to distinguish the accumulation signal during exposure from differences arising from the biological material’s original chemical composition.

In addition, the relative accumulation factor (*RAF*) was calculated [32]:

$$RAF=\frac{C_{exposed}-C_{initial}}{C_{initial}}$$

The *RAF* indicates the extent of enrichment relative to the baseline level and is widely used in active biomonitoring involving mosses, as it allows for comparisons of accumulation between species and elements with different baseline concentrations [6,33]. Negative ΔC and *RAF* values were interpreted as a net lack of accumulation, potential loss, leaching or the effect of analytical variability. In ranking and risk-related analyses, where non-negative values are required, negative ΔC values were treated as a net lack of enrichment.

The ranking of element contributions based on net accumulation was used solely as a tool to organise the relative contributions of individual PTEs to the accumulation signal in mosses. The results of this ranking were not interpreted as a conventional ecological risk assessment or as an assessment of the threat to the study area. Due to differences in the biological matrix of soils and sediments, this ranking was treated as a supplementary analysis to support the interpretation of the accumulation profile [24]. This procedure was used exclusively as an internal accumulation-based ranking of elements and moss species within the present dataset. It does not constitute a classical ecological risk assessment because it does not incorporate toxicity-response coefficients, environmental reference values, exposure pathways, or predicted ecological effects.

The filter and moss datasets were compared at the level of elemental profiles rather than by one-to-one temporal matching. The filters represented discrete 24 h samples, whereas the moss data comprised six cumulative exposure endpoints per species. PCA included normalised profiles of individual filters and species-by-endpoint moss samples. Bray-Curtis dissimilarities were calculated between the campaign-mean normalised PM_10_ profile and the mean normalised profile of each moss species. Absolute concentrations in filters and mosses were not compared directly. This approach is consistent with the assumption that mosses and filters provide complementary information but are not interchangeable methods [5,12].

### 4.6. Statistical Analysis

Descriptive statistics were calculated for PTE concentrations in PM_10_ material collected on filters, net accumulation values (ΔC), *RAF* factors, and risk-related indices calculated for the moss material. For PTE concentrations in the filter material, the minimum, first quartile, median, third quartile, maximum, mean and standard deviation are presented. Particle size descriptors, including Min., Max., Q_10_, Q_50_, Q_90_, Mean, and StdDev, were compiled in the Supplementary Materials and used in the analysis of the relationship with the elemental composition of PM_10_ (Table S1 in the Supplementary Materials).

The relationships between seven particle-size descriptors (Min., Max., Q_10_, Q_50_, Q_90_, Mean, and StdDev) and the concentrations of Cd, Ba, Pb, Cr, Mn, Fe, Ni, Cu, Zn, As, V, and Co in the filter material were assessed using Spearman’s rank correlation. Spearman’s correlation was chosen due to the possible non-normality of the distributions, the presence of outliers, and the expected monotonic, though not necessarily linear, nature of the relationships. For variables showing no variation, correlations were not calculated; such relationships were designated as unestimable. This applied to the minimum value of the particle-size descriptor, which was constant across the analysed dataset.

*RAF* values are presented as arithmetic means across the six cumulative exposure endpoints, separately for each moss species and element. This descriptive use of mean or average *RAF* values is consistent with active moss-bag biomonitoring studies in which *RAF* is used to summarise relative accumulation responses rather than as a variable for parametric inference [6,32]. The error bars represent the standard deviation among the six endpoint-specific mean values. The interpretation of *RAF* was based on the direction and magnitude of relative enrichment, with particular attention paid to the thresholds of 0.5 and 1.0 [21]. The *RAF* thresholds of 0.5 and 1.0 were used as literature-based interpretative reference values following Sergeeva et al. [21], rather than as distribution-derived or statistically tested cut-off points. More broadly, the use of *RAF*, relative accumulation, or enrichment-based descriptors is consistent with active moss-bag biomonitoring studies in which exposed moss material is interpreted through relative changes in element accumulation [8,34,35]. These thresholds were applied consistently to all species and elements to support descriptive interpretation of continuous *RAF* values, not to create a binary enriched/non-enriched classification. Normality tests, confidence intervals, coefficients of variation, and formal significance tests comparing element-specific *RAF* values among moss species were not calculated because the six *RAF* values represented successive cumulative exposure endpoints rather than independent random replicates. Accordingly, *RAF* means and standard deviations were used only as descriptive campaign-level summaries of relative enrichment, not as a basis for inferential comparison among species.

A comparison of the elemental profiles of mosses and filters was carried out using multivariate analyses. The PM_10_ profiles and the accumulation profiles in mosses were compared after normalisation to relative profiles to minimise the influence of differences in units, matrix, and concentration scales. PCA and cluster analysis based on Bray-Curtis distance were used to assess the separation of the profiles. Bray-Curtis distance was used to measure dissimilarity between non-negative, quantitative composition profiles, in line with its use in ecological and environmental analyses [36,37]. The aim of this analysis was not to demonstrate full methodological consistency, but to determine whether PM_10_ and mosses form distinct yet interpretably related PTE profiles.

Statistical calculations were performed using Statistica 13.3, with the R (version 4.1.0)/Python (version 3.11) environment used for auxiliary calculations, profile analyses and the preparation of visualisations. The visualisations included a Spearman correlation heatmap between particle-size descriptors and PM_10_ PTE concentrations, a graph of average *RAF* values over six months of exposure, a PCA analysis, and a Bray-Curtis dendrogram.

## 5. Conclusions

Fe, Zn, and Pb dominated the material retained on PM_10_ filters, while variability in the concentrations of selected elements indicated episodic inputs of particulate matter. Pb provided the clearest concordant signal across both approaches, combining a prominent mass fraction in PM_10_ material with marked relative enrichment in all three moss species, and should therefore be prioritised in further environmental and exposure-oriented monitoring. Particle size may support the interpretation of PTE profiles, but it does not replace information on emission sources, mineral composition, or the chemical forms of the elements.

The accumulation response of mosses depended on both species and element. The use of ΔC and *RAF* made it possible to assess the actual net increase relative to initial concentrations, which is more justified than comparing final concentrations alone. Descriptively, *P. schreberi* showed the broadest profile of relative enrichment; however, the results do not indicate a single universally best biomonitoring species.

The separation of PM_10_ and moss profiles in multivariate analyses confirms that the two matrices record different environmental signals. These differences support the parallel use of both methods because PM_10_ filters provide a short-term record of particle-bound elements, whereas mosses integrate retention and accumulation over the exposure period. Both approaches identified Pb as a priority element, but moss health could not be evaluated because no physiological endpoints were measured.

## Figures and Tables

**Table 1:** Descriptive statistics of PTE concentrations in PM_10_ filter-deposited material.

Element	Min	Q1	Median	Q3	Max	Mean	SD
Cd	0.107	8.58	16.4	51.6	9764	298	1381
Ba	30.7	74.0	130	231	1585	213	254
Pb	198	427	543	1201	4306	911	814
Cr	33.5	49.2	62.5	111	565	109	109
Mn	57.9	172	241	459	1186	329	243
Fe	3475	7464	10,621	18,163	40,847	13,838	9764
Ni	22.7	34.6	43.5	78.0	351	68.2	64.1
Cu	120	173	269	456	1660	374	302
Zn	557	1251	1739	2430	22,184	2753	3933
As	19.6	38.5	49.7	76.8	215	66.8	48.7
V	3.13	10.7	16.2	31.2	66.6	22.3	15.9
Co	3.24	4.82	5.99	11.2	22.2	8.39	5.42

**Table 2:** Accumulation-based contribution ranking of PTEs in mosses.

Sample	Contribution Score	Dominant Contributor	Dominant Value	Second Contributor	Second Value
Dp1	23.5	Zn	8.74	Cu	7.68
Dp2	37.8	Cu	14.9	Zn	10.5
Dp3	73.1	Pb	29.9	Cu	18.6
Dp4	124	Pb	49.5	Cu	33.6
Dp5	116	Pb	41.3	Cu	40.9
Dp6	123	Pb	44.9	Cu	39.4
Sp1	15.8	Zn	10.9	Pb	3.91
Sp2	29.1	Pb	11.8	Zn	9.19
Sp3	78.3	Pb	32.8	Cu	20.5
Sp4	81.8	Pb	33.4	Cu	23.3
Sp5	140	Pb	55.2	Cu	42.7
Sp6	145	Pb	52.0	Cu	46.9
Pl1	48.0	Zn	42.8	Pb	4.59
Pl2	31.4	Zn	16.8	Pb	8.15
Pl3	130	Zn	68.0	Pb	35.5
Pl4	212	Zn	88.1	Pb	55.4
Pl5	156	Pb	51.2	Zn	47.6
Pl6	156	Pb	49.4	Cu	45.4
**Species contribution to net accumulation-based score**					
**Species**	**Total net score**		**Share pct**		
Cr	30.0		1.74		
As	37.9		2.20		
Cd	37.2		2.16		
Ni	57.7		3.35		
Cu	451		26.2		
Dp	497		28.9		
Sp	490		28.5		
Zn	534		31.1		
Pb	572		33.3		
Pl	733		42.6		

## Data Availability

The data presented in this study are available in this study only and in the supporting information in Supplementary Materials.

## Supplementary Materials

The following supporting information can be downloaded at: https://www.mdpi.com/article/10.3390/molecules31132393/s1, Table S1. Descriptive statistics of PM_10_ filters. Table S2. ICP-MS operating and data-acquisition parameters used during the analysis.

## References

[1] Świetlik R., Trojanowska M. (2022). Chemical Fractionation in Environmental Studies of Potentially Toxic Particulate-Bound Elements in Urban Air: A Critical Review. Toxics.

[2] Harrison R.M., Allan J., Carruthers D., Heal M.R., Lewis A.C., Marner B., Murrells T., Williams A. (2021). Non-Exhaust Vehicle Emissions of Particulate Matter and VOC from Road Traffic: A Review. Atmos. Environ..

[3] Piscitello A., Bianco C., Casasso A., Sethi R. (2021). Non-Exhaust Traffic Emissions: Sources, Characterization, and Mitigation Measures. Sci. Total Environ..

[4] Löber M., Bondorf L., Grein T., Reiland S., Wieser S., Epple F., Philipps F., Schripp T. (2024). Investigations of Airborne Tire and Brake Wear Particles Using a Novel Vehicle Design. Environ. Sci. Pollut. Res..

[5] Świsłowski P., Nowak A., Wacławek S., Ziembik Z., Rajfur M. (2022). Is Active Moss Biomonitoring Comparable to Air Filter Standard Sampling?. Int. J. Environ. Res. Public Health.

[6] Urošević Aničić M., Lazo P., Stafilov T., Nečemer M., Andonovska K.B., Balabanova B., Hristozova G., Papagiannis S., Stihi C., Suljkanović M. (2022). Active Biomonitoring of Potentially Toxic Elements in Urban Air by Two Distinct Moss Species and Two Analytical Techniques: A Pan-Southeastern European Study. Air Qual. Atmos. Health.

[7] Rajfur M., Stoica A.I., Świsłowski P., Stach W., Ziegenbalg F., Mattausch E.M. (2024). Assessment of Atmospheric Pollution by Selected Elements and PAHs during 12-Month Active Biomonitoring of Terrestrial Mosses. Atmosphere.

[8] Vázquez-Arias A., Giráldez P., Martínez-Abaigar J., Núñez-Olivera E., Aboal J.R., Fernández J.Á. (2024). Fine-Tuning the Use of Moss Transplants to Map Pollution by Potentially Toxic Elements (PTEs) in Urban Areas. Sci. Total Environ..

[9] García-Seoane R., Antelo J., Fiol S., Fernández J.A., Aboal J.R. (2023). Unravelling the Metal Uptake Process in Mosses: Comparison of Aquatic and Terrestrial Species as Air Pollution Biomonitors. Environ. Pollut..

[10] García-Seoane R., Fernández J.A., Chilà A., Aboal J.R. (2019). Improving the Uptake of Pollutants in Moss Bags: The Wind Effect. Ecol. Indic..

[11] Ares A., Aboal J.R., Carballeira A., Giordano S., Adamo P., Fernández J.A. (2012). Moss Bag Biomonitoring: A Methodological Review. Sci. Total Environ..

[12] Rajfur M., Świsłowski P., Turlej T., Isinkaralar O., Isinkaralar K., Almasi S., Callegari A., Stoica A.I. (2025). Comparative (Bio)Monitoring of Airborne PAHs Using Mosses and Filters. Molecules.

[13] Di Palma A., Capozzi F., Spagnuolo V., Giordano S., Adamo P. (2017). Atmospheric Particulate Matter Intercepted by Moss-Bags: Relations to Moss Trace Element Uptake and Land Use. Chemosphere.

[14] Patel A., Aggarwal S., Bard L., Durif O., Introna M., Juárez-Facio A.T., Tu M., Elihn K., Nozière B., Olofsson U. (2024). Gaseous Emissions from Brake Wear Can Form Secondary Particulate Matter. Sci. Rep..

[15] Kaonga C.C., Kosamu I.B.M., Utembe W.R. (2021). A Review of Metal Levels in Urban Dust, Their Methods of Determination, and Risk Assessment. Atmosphere.

[16] Zioła N., Słaby K. (2020). The Content of Selected Heavy Metals and Polycyclic Aromatic Hydrocarbons (PAHs) in PM10 in Urban-Industrial Area. Sustainability.

[17] Ahmad S., Zeb B., Ditta A., Alam K., Shahid U., Shah A.U., Ahmad I., Alasmari A., Sakran M., Alqurashi M. (2023). Morphological, Mineralogical, and Biochemical Characteristics of Particulate Matter in Three Size Fractions (PM10, PM2.5, and PM1) in the Urban Environment. ACS Omega.

[18] Hu R., Yan Y., Zhou X., Wang Y., Fang Y. (2018). Monitoring Heavy Metal Contents with Sphagnum Junghuhnianum Moss Bags in Relation to Traffic Volume in Wuxi, China. Int. J. Environ. Res. Public Health.

[19] Lanphear B., Navas-Acien A., Bellinger D.C. (2024). Lead Poisoning. N. Engl. J. Med..

[20] Leyssens L., Vinck B., Van Der Straeten C., Wuyts F., Maes L. (2017). Cobalt Toxicity in Humans—A Review of the Potential Sources and Systemic Health Effects. Toxicology.

[21] Sergeeva A., Zinicovscaia I., Vergel K., Yushin N., Urošević M.A. (2021). The Effect of Heavy Industry on Air Pollution Studied by Active Moss Biomonitoring in Donetsk Region (Ukraine). Arch. Environ. Contam. Toxicol..

[22] Macedo-Miranda M.G., Barrera-Díaz C.E., Avila-Pérez P., López-Solórzano E., Ortiz-Oliveros H.B., Zavala-Arce R.E. (2024). Bioconcentration Capacity of Moss Leskea Angustata Tayl., for Heavy Metals and Its Application in the Atmospheric Biomonitoring of a Metropolitan Area. Atmos. Environ..

[23] Schillaci L., Djakovic N., Lang I. (2023). Is a Combination of Metals More Toxic to Mosses Than a Single Metal?. Plants.

[24] Hakanson L. (1980). An Ecological Risk Index for Aquatic Pollution Control a Sedimentological Approach. Water Res..

[25] Sfetsas T., Ghoghoberidze S., Karnoutsos P., Tziakas V., Karagiovanidis M., Katsantonis D. (2025). Urban Source Apportionment of Potentially Toxic Elements in Thessaloniki Using Syntrichia Moss Biomonitoring and PMF Modeling. Environments.

[26] Bukowska P. (2026). Seasonal Characteristics and Key Sources of Trace Element Deposition Fluxes in Coastal Poland. Sci. Rep..

[27] ICP Vegetation (2020). Heavy Metals, Nitrogen and POPs in European Mosses: 2020 Survey.

[28] Świsłowski P., Kosior G., Rajfur M. (2021). The Influence of Preparation Methodology on the Concentrations of Heavy Metals in *Pleurozium Schreberi* Moss Samples Prior to Use in Active Biomonitoring Studies. Environ. Sci. Pollut. Res..

[29] (2002). Digestion for the Determination of Elements in Water—Part 2: Nitric Acid Digestion.

[30] (2004). Application of Inductively Coupled Plasma Mass Spectrometry (ICP-MS)—Part 2: Determination of 62 Elements.

[31] (2009). Guidance on Analytical Quality Control for Chemical and Physicochemical Water Analysis.

[32] Gómez-Ensastegui C., Avila-Pérez P., García-Rivas J.L., Barrera-Díaz C.E., Ortiz-Oliveros H.B., Martínez-Gallegos S. (2025). Evaluation of an Aquatic Liverwort and Terrestrial Moss as Biomonitors of Heavy Metals Associated with Particulate Matter. Sci. Rep..

[33] Culicov O.A., Mocanu R., Frontasyeva M.V., Yurukova L., Steinnes E. (2005). Active Moss Biomonitoring Applied to an Industrial Site in Romania: Relative Accumulation of 36 Elements in Moss-Bags. Environ. Monit. Assess..

[34] Capozzi F., Giordano S., Aboal J.R., Adamo P., Bargagli R., Boquete T., Di Palma A., Real C., Reski R., Spagnuolo V. (2016). Best Options for the Exposure of Traditional and Innovative Moss Bags: A Systematic Evaluation in Three European Countries. Environ. Pollut..

[35] Capozzi F., Adamo P., Di Palma A., Aboal J.R., Bargagli R., Fernandez J.A., Lopez Mahia P., Reski R., Tretiach M., Spagnuolo V. (2017). Sphagnum Palustre Clone vs Native Pseudoscleropodium Purum: A First Trial in the Field to Validate the Future of the Moss Bag Technique. Environ. Pollut..

[36] Legendre P., Legendre L. (2012). Numerical Ecology: Developments in Environmental Modelling.

[37] Borcard D., Gillet F., Legendre P. (2018). Numerical Ecology with R.

