# Supplementary material for: PM10 Filter Monitoring and Moss-Bag Biomonitoring as Complementary Approaches for Assessing Atmospheric Deposition of Potentially Toxic Elements
Source: Molecules. 2026 Jul 7;31(13):2393. doi: 10.3390/molecules31132393 (PMC13363318; doi:10.3390/molecules31132393)
Supplement: Supplementary file 1 [file molecules-31-02393-s001.zip › molecules-4403202-supplementary.pdf]

Supplementary Materials

PM_{10} Filter Monitoring and Moss-Bag Biomonitoring as Complementary Approaches for Assessing Atmospheric Deposition of Potentially Toxic Elements

Paweł Świsłowski [1,*], Małgorzata Rajfur [1], Tymoteusz Turlej [2], Inga Zinicovscaia [3], Oznur Isinkaralar [4], Kaan Isinkaralar [5] and Anca-Iulia Stoica [6]

[1] Institute of Biology, University of Opole, 45-032 Opole, Poland; rajfur@uni.opole.pl
[2] Department of Power Systems and Environmental Protection Facilities, AGH University of Krakow, 30-059 Cracow, Poland; turlej@agh.edu.pl
[3] Horia Hulubei National Institute for R&D in Physics and Nuclear Engineering, 077125 Magurele, Romania; zinikovskaia@mail.ru
[4] Department of Landscape Architecture, Faculty of Engineering and Architecture, Kastamonu University, Kastamonu 37150, Türkiye; obulan@kastamonu.edu.tr
[5] Department of Environmental Engineering, Faculty of Engineering and Architecture, Kastamonu University, Kastamonu 37150, Türkiye; kisinkaralar@kastamonu.edu.tr
[6] Institute of Sanitary Engineering and Water Pollution Control (SIG), University of Natural Resources and Life Sciences, 1190 Vienna, Austria; anca.stoica@boku.ac.at
* Correspondence: pawel.swislowski@uni.opole.pl

Table S1. Descriptive statistics of PM_{10} filters

Descriptor	min	Q1	median	Q3	max	mean	SD
Min.	0.160	0.160	0.160	0.160	0.160	0.160	0.000
Max.	28.7	38.1	45.3	59.1	68.3	47.6	12.6
Q10	0.170	0.170	0.170	0.175	0.230	0.177	0.015
Q50	0.210	0.250	0.270	0.410	1.64	0.386	0.292
Q90	0.430	0.950	1.13	1.63	4.48	1.33	0.797
Mean	0.490	0.625	0.700	0.745	1.08	0.718	0.147
StdDev	0.820	0.980	1.07	1.21	1.49	1.10	0.158

Table S2. ICP-MS operating and data-acquisition parameters used during the analysis

Parameter	Setting
Instrument	PerkinElmer NexION 2000 ICP-MS
RF power	1550 W
Auxiliary gas flow	1.2 mL min−1
Internal standard	Indium
Internal-standard addition	0.5 mL of 100 µg L−1 In solution per 5 mL sample
Sweeps per reading	20

Parameter	Setting
Readings per replicate	1
Replicates per sample	3
Integration time	1000 ms amu−1
Approximate method time	2 min 46 s per sample
Reading time	35.64 s
Replicate time	35.64 s
Pressurisation delay	30 s
Exhaust delay	0 s
Channel delay	0 s
Settling delay	0 s
Flow switching delay	30 s
Monitored isotopes	111Cd, 138Ba, 208Pb, 52Cr, 55Mn, 56Fe, 60Ni, 65Cu, 66Zn, 75As, 51V, 59Co, 202Hg
